# The Efficiency of Lemon Essential Oil-Based Nanoemulsions on the Inhibition of *Phomopsis* sp. and Reduction of Postharvest Decay of Kiwifruit

**DOI:** 10.3390/foods11101510

**Published:** 2022-05-22

**Authors:** Fan-Bing Meng, Zhen-Zhen Gou, Yun-Cheng Li, Long-Hua Zou, Wei-Jun Chen, Da-Yu Liu

**Affiliations:** 1 College of Food and Biological Engineering, Chengdu University, Chengdu 610106, China; mfb1020@163.com (F.-B.M.); gouzhenzhen@163.com (Z.-Z.G.); zoulonghua@cdu.edu.cn (L.-H.Z.); cwj19860417@163.com (W.-J.C.); 2 Key Laboratory of Coarse Cereal Processing, Ministry of Agriculture and Rural Affairs, Chengdu University, Chengdu 610106, China; liudy1014@163.com

**Keywords:** lemon essential oil, nanoemulsion, *Phomopsis* sp., inhibitory effect, kiwifruit, preservation

## Abstract

Essential oils (EOs) have excellent antibacterial activity and are generally recognized as safe (GRAS) for use in food preservatives. However, the application of EOs is limited because of their strong volatility and easily oxidized. Encapsulation of EOs into nanoemulsions could effectively prevent oxidative deterioration. In this study, lemon essential oil-based nanoemulsion (LEO/NE) was prepared by high-pressure homogenization. FT-IR and encapsulation efficiency analysis indicated that LEO was effectively encapsulated in the nanoemulsion. The results of zeta potential changes after 35 d storage indicated that LEO/NE exhibits good stability at room temperature. The effect of LEO/NE on the main soft rot pathogens of kiwifruit *Phomopsis* sp. was investigated, and the results showed that LEO/NE significantly inhibited spore germination and mycelia growth of *Phomopsis* sp. by promoting ROS accumulation, intracellular antioxidant enzyme activities, and cell apoptosis. The preservation experiment was carried out by inoculating *Phomopsis* sp. spores into fresh kiwifruit, and the LEO/NE effectively inhibited soft rot development in kiwifruit in a LEO dose dependent manner. LEO/NE with 1% LEO loading amount has a good effect on preventing postharvest decay of kiwifruit caused by *Phomopsis* sp.

## 1. Introduction

Kiwifruit (*Actinidia* spp.) is a juicy, nutritious and delicious climacteric fruit with typical berry characteristics and thin skin [1], so it has a short shelf life and is highly perishable after harvest, which greatly restricts market circulation and leads to a large number of unsalable fruit and a backlog of defective fruit, causing huge economic losses [2]. Postharvest diseases caused by pathogens, such as *Phomopsis* sp., *Botryosphaeria dothidea*, *Botrytis cinerea*, etc., are the most serious challenge for kiwifruit preservation and have resulted in decay and a decrease in quality, shelf life, and postharvest losses [3]. According to previous report, the loss rate of kiwifruit caused by postharvest diseases is as high as 40% in Guizhou province of China [4]. Among these pathogens, *Phomopsis* sp. is one of the major fungal genus that can cause soft rot in kiwifruit [5]. It can enter kiwifruit tissues and remain latent there until the fruit ripens, and then begin to recover their infectious capacity, eventually causing fruit rot symptoms during storage [3]. At present, the storage and preservation of kiwifruit mostly focuses on physical low temperature and chemical fungicides preservatives [6,7], but increased concerns for food safety and environmental risks have led to the development of new alternative approaches to disease control [8]. In this context, green preservation technologies based on plant extracts have attracted much attention in recent years [9]. For example, a composite film containing chitosan, dextrin, ferulic acid, calcium, and auxiliaries was proven it has a good effect on inhibiting the occurrence of soft rot caused by *Phomopsis* sp. during field growth of kiwifruit [3].

Plant essential oils (EOs) refer to the volatile aromatic substances extracted from the flowers, leaves, stems, roots, or fruit of plants [10]. EOs were shown to have a broad spectrum of antimicrobial and antifungal activities because they contain terpenoids, especially monoterpenes (C10), sesquiterpenes (C15), and diterpenes (C20), along with a variety of aliphatic hydrocarbons (low molecular weight), phenols, acids, alcohols, aldehydes, and esters [11,12]. Currently, many EOs and their ingredients are approved by the European Commission (EC) and the U.S. Food and Drug Administration (FDA), which classifies these natural ingredients and mixtures to be generally recognized as safe (GRAS) for use in food preservatives to reduce or replace the use of chemical preservatives [13]. However, EOs are volatile, easily oxidized, highly hydrophobic, and have a unique taste, which greatly limits their application. Previous studies have shown that the encapsulation of EOs into nanoemulsions could effectively prevent their oxidative deterioration [14,15]. On the other hand, encapsulation could achieve the purpose of slow release to prolong the action time, and at the same time, it will also significantly enhance the antimicrobial properties, which is attributed to the fact that EOs more easily enter microbial cells, so the preparation of essential oil nanoemulsions can greatly promote the practical application of essential oils as green food preservatives [13,16].

Lemon essential oil (LEO) is mainly extracted from lemons by cold pressing or steam distillation. It consists of more than 200 compounds, more than 85% of which are volatile compounds. LEO mainly contains oxygenated compounds such as alcohols, aldehydes, ketones, esters, and terpenoids, which have anticancer, antioxidant, antiviral, anti-inflammatory and bactericidal effects [17,18]. Thus, LEO has been developed into a green preservative and applied in meat and fruit storage to prevent oxidation and spoilage of bacteria [18,19]. Although a previous study showed that LEO has a good inhibitory effect on foodborne fungi [20], the effects of LEO on soft rot fungi of kiwifruit are rarely reported. Therefore, in the present study, a LEO-based nanoemulsion was prepared by a low-esterification modified konjac glucomannan octenyl succinate (KGOS), an excellent-performance nanocapsule wall material developed in a previous study [21], and its antifungal activities against the kiwifruit pathogen *Phomopsis* sp. and reduction of postharvest decay in kiwifruit were investigated.

## 2. Materials and Methods

### 2.1. Materials

Mature Jinyan kiwifruit (*Actinidia chinensis×Actinidia eriantha*) were picked from the orchard, and then transported to a local supermarket within 24 h. The kiwifruit with similar shape and weight (70 g ± 5 g) without rot or physical injuries were chosen for the following experiments. LEO, extracted from Eureka lemon (*Citrus limon*) peel by distillation, was obtained from Lvyuan Lemon Development Co., Ltd. (Sichuan, China). The fungal pathogen *Phomopsis* sp. (Bio-21879) originating from kiwifruit was obtained from Biobw Biotechnology Co., Ltd. (Beijing, China). It was cultured at 25 °C on potato dextrose agar (PDA) for one month. Conidia were collected and suspended in sterile distilled water and filtered through two layers of sterile cheesecloth to remove mycelia.

The Bradford protein assay kit and ROS detection kit were purchased from Shanghai Biyuntian Biotechnology Co., Ltd. (Shanghai, China). Ultratrace total ATPase detection kits, T-SOD assay kits, CAT assay kits, and GSH-PX assay kits were purchased from Nanjing Jiancheng Bioengineering Institute (Nanjing, China). An Annexin V- Alexa Fluor 488/PI kit was purchased from Beijing Soleibo Technology Co., Ltd. (Beijing, China).

### 2.2. Determination of the Chemical Constituents of LEO

The chemical constituents of the LEO (10 times diluted by *n*-hexane) were detected by gas chromatography—mass spectrometry (GC/MS) (QP2010, Shimadzu, Kyoto, Japan) using the method described previously with some modifications [14]. Chemical compounds were separated using a Stabilwax column (60 m × 0.25 mm i.d. × 0.25 μm). Helium was used as the carrier gas at a constant flow rate of 1 mL/min. The oven initial temperature was 40 °C. The temperature increased to 180 °C at a rate of 20 °C/min, maintained for 1 min and finally increased to 230 °C at 20 °C/min, then maintained for 15 min. The pressure was 49.5 kPa, the flow rate was 1.0 mL/min, and the injection volume was 1 μL.

Chemical compounds were identified by comparing their retention indices (RIs) and mass spectra with those in the data system library of the National Institute of Standards and Technology (NIST14). C_7_–C_40_ *n*-alkanes (Sigma, St. Louis, MO, USA) were run under the same conditions and applied as standard references to calculate retention index (RI) values. The relative percentage for each compound present in the essential oil was determined based on chromatographic peak areas. The measurements were performed in triplicate.

### 2.3. Preparation of LEO Nanoemulsions

Konjac glucomannan octenyl succinate (KGOS) was prepared using a microwave method as described previously [22]. Briefly, 20 g of KGM powder (dry weight) and 0.4 g of Na_2_CO_3_ were added to a reaction vessel. Twenty grams of ethanol solution (30%) and 3% OSA (*w*/*w* in proportion to KGM) were added slowly with agitation. The mixture was microwave reacted at 300 W and 70 °C for 20 min, blended with 40 mL of ethanol solution (30%) for 5 min, and adjusted pH to 6.50 with HCl solution (1 N). The mixture was washed with 30 % ethanol and absolute ethanol five times, respectively, to remove residual impurities. The final solid material was dried and passed through a 100-mesh nylon sieve. The KGOS with an SR of approximately 1.539% was selected for this study.

LEO nanoemulsion (LEO/NE) was prepared according to our previous study with minor modifications [23]. A certain amount of KGOS was weighed and dissolved in water to create a 0.4% solution, and 0.8% Tween 80 and different amounts of LEO were added. The mixture was dispersed on a high-speed disperser at 12,000 r/min for 1 min, and then homogenized for 90 s using high-pressure homogenizer (SRH 60–70, Samro Homogenizer Co., Ltd., Shanghai, China) at 30 MPa.

### 2.4. Characterization of LEO/NE

#### 2.4.1. Detection of the Encapsulation Efficiency

The encapsulation efficiency of LEO in the nanoemulsion was mainly evaluated by detecting the content of the main antibacterial component limonene [24]. Limonene was determined by a high-performance liquid chromatograph (HPLC) (1260, Angilent, Santa Clara, CA, USA) equipped with a diode array detector (DAD) and a BDS C18 column (5 mm, 250 × 4.6 mm, Thermo Fisher, Austin, USA) maintained at 30 °C. The mobile phase was methanol-acetonitrile-water (63:22:15) at a flow rate of 1.2 mL/min during the 20 min of analysis. The injection volume was 20 μL, and the samples were previously filtered through a 0.2 μm filter. The encapsulation efficiency was estimated by centrifuging the nanoemulsion suspensions at 5000× *g* for 10 min and analyzing the supernatant without further dilution. The encapsulation efficiency (EE) was calculated using Equation (1) [13].

(1) $$\mathrm{EE}\left(\%\right)=\frac{C_{t}-C_{f}}{C_{t}}\times 100$$

where C_t_ is the total limonene concentration in the nanoemulsion formulation and C_f_ is the limonene concentration in the nanoemulsion suspension supernatant. All measurements were performed in triplicate.

#### 2.4.2. Dynamic Light Scattering (DLS) and Zeta Potential (ZP)

The particle size, polydispersity index (PDI) and zeta potential of the nanoemulsion at room temperature for 0 d and 35 d were determined based on dynamic light scattering (DLS) measurements made using a Zetasizer Nano ZEN3600 instrument (Malvern Instruments Ltd., Malvern, UK) equipped with a He-Ne laser (633 nm) at 25 °C and 90° collecting optics. All measurements were performed in triplicate. The size and zeta potential results are expressed in nm and millivolts, respectively [25].

#### 2.4.3. Fourier Transform Infrared (FT-IR) Spectroscopy Analysis

The nanoemulsions with a LEO loading amount of 1.5% (*w*/*w*) were lyophilized before FT-IR analysis. The samples of LEO, LEO/NE, and wall materials (Wm) were ground further and mixed with KBr. Each sample (3 mg) was loaded onto a Spectrum Two FT-IR spectrometer (PerkinElmer, Massachusetts, USA). The infrared spectra were collected from 400 to 4000 cm^−1^. Each sample was subjected to 60 repeated scans [26]. All measurements were performed in triplicate.

### 2.5. Effects of LEO/NE on Spore Germination of the Fungal Phomopsis *sp*.

The effects of LEO/NE on spore germination of the fungal *Phomopsis* sp. were determined according to the method by Zhang et al. [27] with some modifications. A certain amount of LEO/NE was added to the potato dextrose broth (PDB) medium to final concentrations of LEO of 0%, 0.05%, 0.10%, and 0.20%. Pipetted 190 μL of the above medium was inoculated into a 96-well plate, and 10 μL of *Phomopsis* sp. spore suspension (1 × 10^6^ spores/mL) was added to each well. After culturing at 25 °C for 12 h, the spore germination rate was calculated by counting the germination of 200 spores in each replicate. Additionally, after culturing for 2 d, the optical density at 600 nm (OD600) was determined using a microplate reader (H1M, Guangzhou Darui Biotechnology Co., Ltd., Guangzhou, China) to further measure spore germination. Spore germination was checked microscopically (PH2000, Phoenix Optical Holdings Co., Ltd., Jiangxi, China) to confirm the spectrophotometric data. All experiments were performed in triplicate.

### 2.6. Effects of LEO/NE on the Cell Viability and Mycelial Growth of Phomopsis *sp*.

A certain amount of LEO/NE was added to the PDB medium to final concentrations of LEO of 0%, 0.05%, 0.10%, and 0.20%. Pipetted 190 μL of the above medium were inoculated into a 96-well plate, and 10 μL of *Phomopsis* sp. spore suspension (1 × 10^6^ spores/mL) was added to each well. After culturing for 2 d, the mycelia were collected and ultrasonically disrupted by a JY92-IIN ultrasonic cell pulverizer (Scient Z Biotechnology Co., Ltd., Ningbo, China) under 130 W for 3 s, which was repeated 30 times with an interval of 10 s. The protein was extracted according to the manufacturer’s instructions for the Bradford protein assay kits. The protein concentration was determined using the Bradford method with bovine serum albumin as the standard. The ATP level of the mycelia was determined by an Ultra-trace total ATPase detection kit according to the manufacturer’s instructions. The absorbance at 636 nm was recorded [28].

For colony diameter measurements, 30 mL of PDA solid medium was poured into a Petri dish (100 mm in diameter), a certain amount of LEO/NE (25 μL, 50 μL, or 100 μL) was evenly coated onto the Petri dish, and then 50 μL of *Phomopsis* sp. spore suspension (1 × 10^6^ spores/mL) was dropped onto the center of the Petri dish. The colony diameter was recorded at 4 d after inoculation. All of the above experiments were performed in triplicate.

### 2.7. Effects of LEO/NE on Reactive Oxygen Species (ROS) Accumulation of Phomopsis *sp*.

A total of 190 μL of PDB medium was accurately pipetted into a 96-well plate, and 10 μL of *Phomopsis* sp. spore suspension (1 × 10^6^ spores/mL) was inoculated in each well. After culturing for 2 d at 25 °C, a certain amount of LEO/NE was added to the PDB medium to final concentrations of LEO of 0%, 0.05%, 0.10%, and 0.20%. After culturing at 25 °C for 24 h, mycelia were collected for determination of ROS accumulation, apoptosis, and antioxidant enzyme activities. ROS were detected using a ROS detection kit according to the manufacturer’s instructions using the fluorescent probe DCFH-DA. Fluorescence intensity was detected after staining using confocal scanning laser microscopy (CSLM) (FV1200MPE/FV1200, Olympus, Tokyo, Japan) with excitation at 488 nm and emission at 520 nm according to a previous study [22].

### 2.8. Effects of LEO/NE on Intracellular Antioxidant Enzyme Activities in Phomopsis *sp*.

A certain amount of LEO/NE was added to the PDB medium to final concentrations of LEO of 0%, 0.05%, 0.10%, and 0.20%. A total of 4.75 mL of the above medium was accurately pipetted into a 6-well plate and inoculated with 250 μL of *Phomopsis* sp. spore suspension (1 × 10^6^ spores/mL) in each well. After culturing at 25 °C for 2 d, the mycelia were collected and ultrasonically disrupted according to the description of Section 2.6. The protein was extracted according to the manufacturer’s instructions for the Bradford protein assay kits, and the protein concentration was determined using the Bradford method with bovine serum albumin as the standard. The activities of catalase (CAT), superoxide dismutase (SOD) and glutathione peroxidase (GSH-PX) were determined using a T-SOD assay kits, CAT assay kits and GSH-PX assay kits, respectively, according to the manufacturer’s instructions. All of the above experiments were performed in triplicate.

### 2.9. Effects of LEO/NE on Cell Apoptosis of Phomopsis *sp*.

An Annexin V-Alexa Fluor 488/PI kit was used to determine apoptosis. Mycelia collection was the same as described in Section 2.7. Sample preparation and staining were performed according to the manufacturer’s instructions. Green fluorescence (Annexin V-Alexa Fluor 488) was detected with excitation at 488 nm and emission at 520 nm, and red fluorescence (PI) was detected with excitation at 543 nm and emission at 630 nm using an FV1200 MPE/FV1200 CLSM (Olympus, Tokyo, Japan). All of the above experiments were performed in triplicate.

### 2.10. Effect of LEO/NE on the Postharvest Decay of Kiwifruit

The amount of wall material added did not change, and nanoemulsions with different essential oil contents (0.25%, 0.50%, 0.75%, 1%, and 1.25%) were prepared according to the method in Section 2.3. The kiwifruit was surface disinfected with 2% sodium hypochlorite for 3 min, rinsed with sterile water and air-dried. Then, the fruit was immersed with LEO/NE and wall materials (Wm control). After air-drying, *Phomopsis* sp. spore suspension was evenly sprayed (1 × 10^6^ spores/mL) on the surface of the fruit, which were placed in a plastic box (140 mm × 100 mm × 65 mm) and stored at 22 °C with 80% relative humidity for 12 d. The samples without Wm and LEO/NE treatment were assigned as the blank. The incidence of kiwifruit rot was calculated using Equation (2) [28].

(2) $$\mathrm{Incidence}\;\mathrm{of}\;\mathrm{kiwifruit}\;\mathrm{rot}\;\left(\%\right)=\frac{\mathrm{Number}\;\mathrm{ofinnfected}\;\mathrm{fruit}}{\mathrm{Total}\;\mathrm{fruit}\;\mathrm{per}\;\mathrm{replicate}}\times 100$$

All kiwifruit peels were removed, and photographs were taken. The peeled kiwifruit was cut into 0.8 cm thick slices, and the hardness, viscosity, elasticity, chewiness, soluble solid content, and titratable acid were determined according to Chen et al. [29] Seven fruitsfruit were processed for each treatment, and each treatment was performed in triplicate

### 2.11. Statistical Analysis

The data are reported as means ± standard deviations (SD). Statistical computations and analyses were conducted using SPSS 20.0 (IBM Analytics, Tulsa, USA). Data were subjected to analysis of variance (ANOVA), and Tukey’s HSD test was carried out to compare the means. Differences were considered significant at *p* < 0.05 and are represented by different lowercase letters.

## 3. Results and Discussion

### 3.1. Chemical Compositions of LEO

The constituents of LEO based on GC/MS analysis are shown in Table 1. A total of 25 main components were identified, accounting for 99.99% of the LEO components. They are mainly terpenes, aldehydes, esters, and other oxygen-containing compounds. Among them, D-limonene is the chemical component with the highest content of LEO, accounting for 66.51%. Other main components are β-pinene (12.5%), γ-terpinene (9.75%), sabinene (2.03%), α-pinene (1.91%), β-myrcene (1.47%), and *cis*-citral (1.43%). These values are consistent with those published in other studies [19,30]. D-Limonene is a natural antibacterial agent that inhibits the growth of a wide range of bacteria and fungi, including activity against common postharvest fungal pathogens of fruit [31]. Some previous studies indicated that D-limonene coatings can effectively improve meat preservation and limit fruit decay [14,32]. The α- and β-isomers of pinene have also been confirmed to exhibit microbicidal activity against fungi and bacteria; γ-terpinene has good antibacterial, anti-inflammatory and antioxidant effects [33]. Therefore, the chemical compositions of LEO confer antibacterial effects, which enable the feasibility of applying LEO microcapsules to the preservation of kiwifruit.

### 3.2. Physical and Chemical Characteristics of LEO/NE

In the present study, LEO/NE was prepared using high-pressure homogenization under 30 MPa pressure. The average particle size and PDI value of LEO/NE were 163.13 ± 7.74 nm and 0.369 ± 0.020, respectively, and the EE was above 99%, which indicated that the method in this study could be used to produce a stable LEO/NE dispersion with a relatively narrow size distribution. The zeta potential of the LEO/NE is −24.60 ± 1.32 mV. The lower potential helps the molecules repel each other, ensures their stability, and prevents particle aggregation [34]. After LEO/NE was stored for 35 d at room temperature, although the average particle size of LEO/NE was 263.88 ± 33.13 nm and the PDI was 0.409 ± 0.047. Although the zeta potential of LEO/NE decreased to −19.30 ± 1.49 mV, it close to 20 mV, which indicated that LEO/NE still had good stability [35].

### 3.3. FT-IR Spectroscopy Detection of LEO/NE

**Figure 1:**
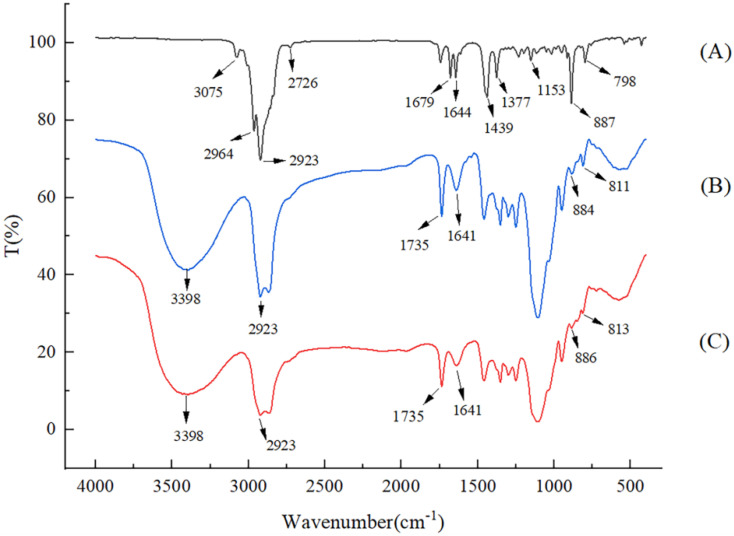
Infrared spectrum of freeze-dried samples of LEO (**A**), KGOS (**B**), and LEO/NE (**C**).

The FT-IR spectroscopy results for the LEO, LEO/NE, and wall material KGOS samples are shown in Figure 1. As shown in Figure 1A, the absorption band at 3100–3000 cm^−1^ was assigned to the stretching vibrations of C-H groups in LEO [32]. The bands at 2964, 2923, and 2858 cm^−1^ resulted from the axial strain vibrations of the C-H bond in aliphatic molecules. The bands at 1670 cm^−1^ corresponding to C-C stretching due to the presence of a citral compound [36]. The band at 1644 cm^−1^ corresponds to the C=C bond. The moderately intense bands at 1439 and 1377 cm^−1^ correspond to the -CH_3_ bond. The band between 1154 and 798 cm^−1^ corresponds to the C-O bonds of alcohols and carboxylic acids, which belong to some minor compounds present in LEO [13].

The spectrum of KGOS shows that the absorption band at 3398 cm^−1^ is assigned to the stretching of methyl -OH, [23] the stretching peak at 2923 cm^−1^ is assigned to methyl-CH, and the peak at 1735 cm^−1^ is assigned to the stretching of acetyl and octenyl succinic anhydride carbonyl (C=O). [26] The absorption peaks at 811 and 884 cm^−1^ correspond to the stretching vibrations of the mannose unit [37]. The above peaks can be observed in the spectrum of LEO/NE. Some main peaks (3075, 2964, 2858, 1679, 1153 cm^−1^) in LEO were invisible or significantly weakened in the spectrum of LEO/NE, and some peaks (2923, 1644, 1439, 1377, 887, 798 cm^−1^) were slightly weaker in LEO/NE (Figure 1C). Compared with KGOS, LEO/NE had no new observed peaks, which indicated that LEO and KGOS were connected to each other inside the molecules without chemical interaction. These phenomena indicate that LEO is embedded in the nanoemulsion and present in LEO/NE with little or no free molecular form.

### 3.4. Effects of LEO/NE on Spore Germination of Phomopsis *sp*.

**Figure 2:**
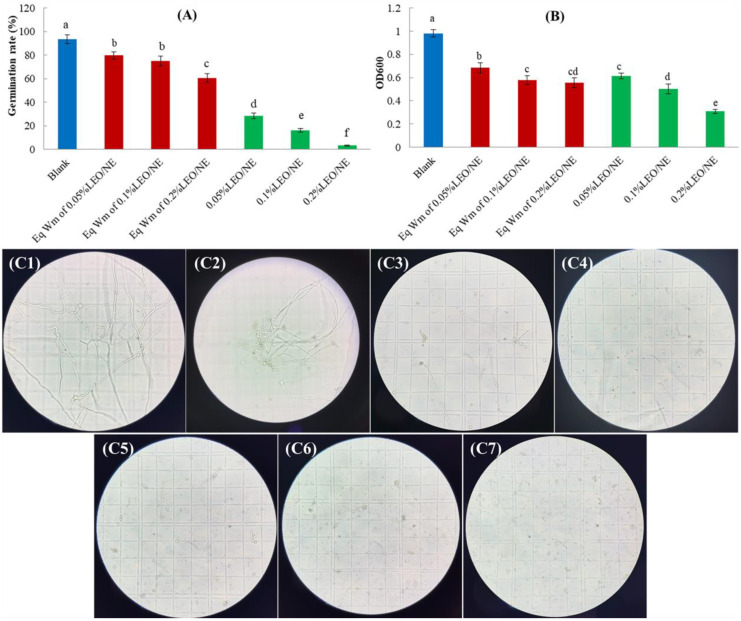
Spore germination of *Phomopsis* sp. under the treatment of lemon essential oil nanoemulsion (LEO/NE) and equivalent wall material (Eq Wm). (**A**) spore germination rate; (**B**) optical density at 600 nm (OD600); (**C1**–**C7**) was microscopic observation of the spore germination at treatment of Blank, Eq Wm of 0.05%LEO/NE, Eq Wm of 0.1%LEO/NE, Eq Wm of 0.2%LEO/NE, 0.05%LEO/NE, 0.1%LEO/NE and 0.2%LEO/NE, respectively. Different letters above each column of (**A**,**B**) indicate significant differences.

As shown in Figure 2, compared to the blank group (without any nanoemulsion treatment), the nanoemulsion prepared by equivalent wall material (Eq Wm) could decrease spore germination. Glucomannan (KGM) is a promising biofilm preservative for fresh fruit and vegetable quality, and this natural product is considered a good candidate for commercial preservation [38]. Konjac glucomannan octenyl succinate (KGOS), which basically retains the properties of konjac glucomannan, has good hydrophilic and lipophilic properties [23]. To further increase the surface activity and stabilize the emulsion for long durations, Tween 80 was added at the same time. The results shown in Figure 2 indicate that the combined use of the two materials manifests a certain inhibitory effect on the spore germination and cell growth of *Phomopsis* sp. However, compared to the equivalent wall material (Eq Wm) treatment, LEO/NE further significantly inhibited the spore germination of *Phomopsis* sp. With the increase in LEO/NE, the spore germination rate gradually decreased. Among the results, when the concentration of LEO was 0.20%, the spore germination rate was only 2.64%, and the inhibition effect was the best. This conclusion could also be proven by the effect of LEO/NE on the spore germination of *Phomopsis* sp. As shown in Figure 2B, with the increase in LEO/NE, the OD600 value gradually decreased, and the difference between different concentrations was significant (*p* < 0.05). Among them, when the concentration of LEO was 0.20%, the OD600 value was 0.309, and the inhibitory effect was the best.

Figure 2C more intuitively shows that the addition of LEO/NE significantly inhibited the spore germination of *Phomopsis* sp. With the increase in the concentration of LEO/NE, the number of germinated spores and length of the germ tube gradually decreased, indicating that LEO/NE could effectively inhibit the spore germination of *Phomopsis* sp. LEO has previously been reported to have antibacterial activity. The study of Ammad et al. [39] reported for the first time the antifungal activity of LEO against three pathogenic fungi that attack vines, and the study by Li et al. [18] reported that LEO can effectively inhibit the growth of *E. coli* during storage. Recently, Kodituwakku et al. [40] compared the inhibitory effects of essential oils from different sources, and Cinnamon bark oil showed the most effects against *Phomopsis* sp. of mango fruit. The present study is the first to focus on the inhibitory effect of LEO nanoemulsions on spore germination of *Phomopsis* sp.

### 3.5. Effects of LEO/NE on Cell Viability and Mycelial Growth of Phomopsis *sp*.

**Figure 3:**
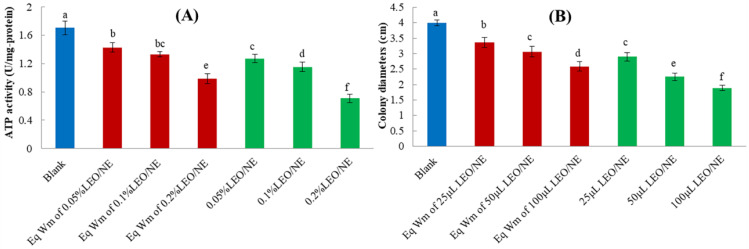
The effect of lemon essential oil nanoemulsion (LEO/NE) and equivalent wall material (Eq Wm) on the intracellular ATPase (**A**) and colony diameters (**B**) of *Phomopsis* sp. Different letters above each column of indicate significant differences.

ATPase is a protease on biological membranes, and it produces the common energy currency in the metabolism of organismal cells. It plays important roles in material transportation, energy conversion, and information transmission [41]. ATP is primarily produced by mitochondrial respiration, and its levels are tightly controlled by the energy regulatory network responsible for ATP synthesis, transport, and consumption. In this network, ATP synthase is the key enzyme in ATP biosynthesis [42]. The hyphae were collected after treatment with Wm and LEO/NE on *Phomopsis* sp. for 2 d to evaluate the effect of LEO/NE on mycelial activity. As shown in Figure 3A,B, with increasing concentrations of Wm, the intracellular ATPase activity decreased, and the colony growth of *Phomopsis* sp. slowed. These results indicated that Wm also has a certain inhibitory effect on *Phomopsis* sp. growth. However, LEO/NE further significantly inhibited the intracellular ATPase activities of *Phomopsis* sp. compared to the Eq Wm treatment, and as the LEO/NE concentration increased, the ATPase activity of mycelium gradually decreased. The effect of LEO/NE on the colony growth of *Phomopsis* sp. hyphae was evaluated by measuring the colony diameter after treatment with LEO/NE for 4 d. As shown in Figure 3B, after 4 d of cultivation, with the increase in the amount of LEO/NE added, the diameter of the mycelium significantly decreased. The above results indicated that LEO/NE had a significant effect on promoting senescence and inhibiting the mycelial growth of *Phomopsis* sp.

### 3.6. Effects of LEO/NE on Reactive Oxygen Species (ROS) Accumulation of Phomopsis *sp*.

**Figure 4:**
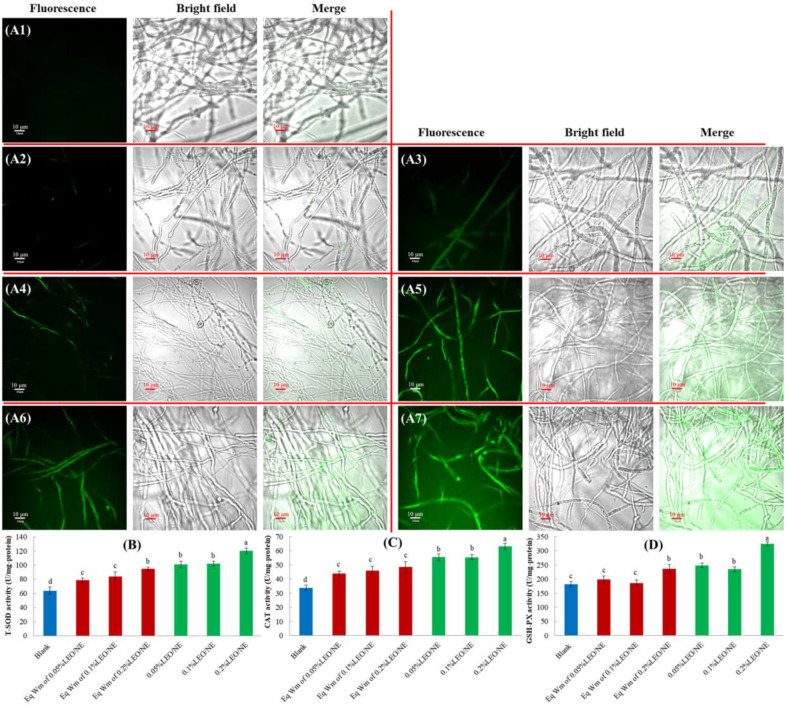
Effects of lemon essential oil nanoemulsion (LEO/NE) and equivalent wall material (Eq Wm) on reactive oxygen species (ROS) accumulation and intracellular antioxidant enzyme activities of *Phomopsis* sp. (**A1**–**A7**) was the DCFH-DA staining results of Blank, Eq Wm of 0.05%LEO/NE, Eq Wm of 0.1%LEO/NE, Eq Wm of 0.2%LEO/NE, 0.05%LEO/NE, 0.1%LEO/NE and 0.2%LEO/NE, respectively; (**B**) CAT activities; (**C**) SOD activities; (**D**) GSH-PX activities. Different letters above each column of (**B**–**D**) indicate significant differences.

Because H_2_O_2_ is the most stable form of ROS, [43] the accumulation of H_2_O_2_ was assessed to measure the level of ROS in hyphal cells using 2′,7′-dichlorodihydrofluorescein diacetate (H_2_DCFDA) staining [27]. The main source of ROS is the mitochondrial respiratory chain of cells. After mitochondria are stimulated by the outside world, the level of cellular ROS increased [44]. As shown in Figure 4A, LEO/NE stimulated the increase of ROS level of *Phomopsis* sp. hyphae, which can induce a significant increase in ROS when the concentration of LEO reaches 0.20%. The results showed that when the LEO/NE gradually increased, it induced an increase in the level of ROS in the hyphae of *Phomopsis* sp. [45].

### 3.7. Effects of LEO/NE on Intracellular Antioxidant Enzyme Activities of Phomopsis *sp*.

CAT, SOD, and GSH-PX activities are often considered indicators of oxidative stress and are stimulated by the increase in ROS levels. Oxidative stress, an imbalance between ROS production and antioxidant systems, has been identified as a common mechanism of cellular damage [46]. CAT, SOD, and GSH-PX activities were determined in hyphae of *Phomopsis* sp. After exposure for 2 d, CAT activities significantly increased (*p* < 0.05) in response to increasing LEO/NE (Figure 4C). SOD and GSH-PX activities were also enhanced (*p* < 0.05) by LEO/NE in a concentration-dependent manner. Activation of these antioxidant enzymes is likely due to the increased levels of ROS produced by LEO microencapsulation, representing an attempt at regulation by *Phomopsis* sp. [44].

### 3.8. Effects of LEO/NE on Cell Apoptosis of Phomopsis *sp*.

**Figure 5:**
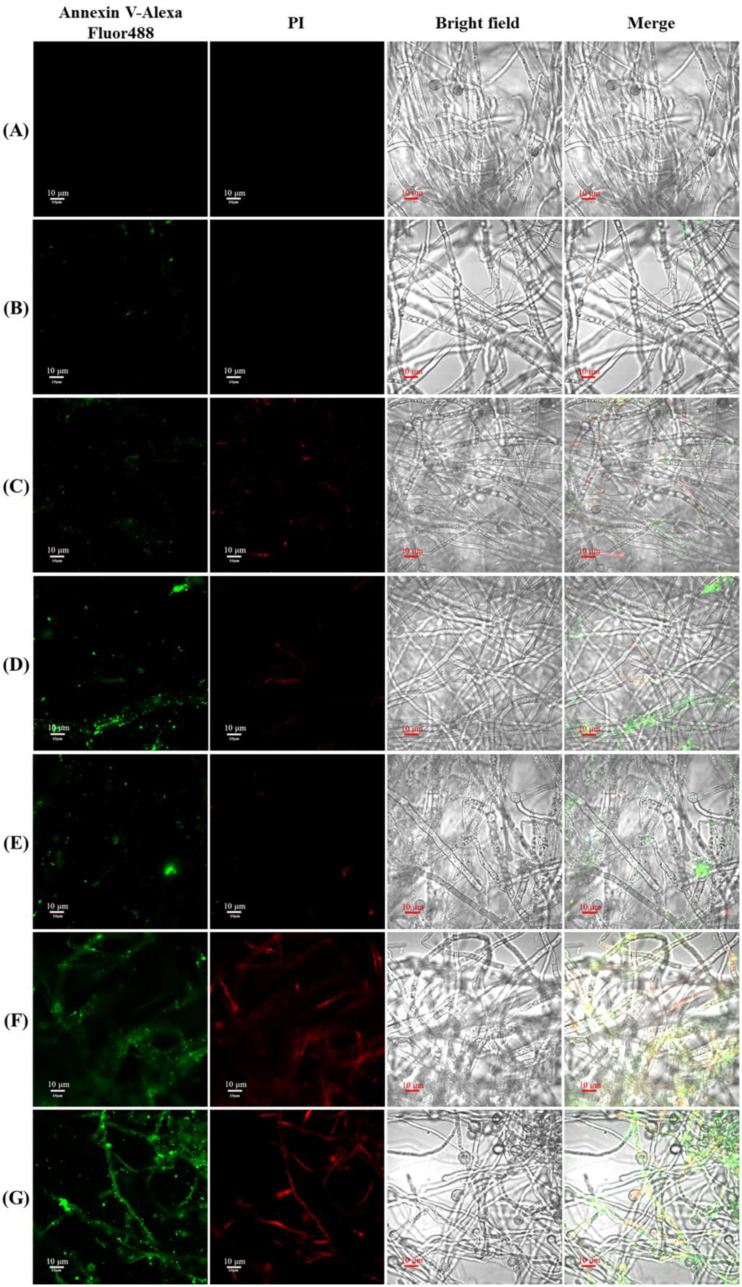
Effects of lemon essential oil nanoemulsion (LEO/NE) and equivalent wall material (Eq Wm) on cell apoptosis of *Phomopsis* sp. Apoptosis was observed using Annexin V-Alexa Fluor 488/PI staining. Fluor 488 positive (green) indicates cell undergone apoptosis. Fluor 488 positive and PI negative (red) indicates cell apoptosis at early stage. Double-positive staining indicates late apoptosis or necrosis. (**A**–**G**) was the double staining results of Blank, Eq Wm of 0.05%LEO/NE, Eq Wm of 0.1%LEO/NE, Eq Wm of 0.2%LEO/NE, 0.05%LEO/NE, 0.1%LEO/NE and 0.2%LEO/NE, respectively; (**B**) CAT activities; (**C**) SOD activities; (**D**) GSH-PX activities.

After treating the hyphae of *Phomopsis* sp. with LEO/NE for 24 h, the effects of LEO/NE on *Phomopsis* sp. cell apoptosis were observed by the Annexin V-Alexa Fluor 488/PI double staining method. Hyphal staining by Annexin V-Alexa Fluor 488 (green) indicated that the cells had undergone apoptosis. The hyphal staining with Annexin V-Alexa Fluor 488 and unstained by PI (red) indicated that the cells were in the early stage of apoptosis. When hyphal double-positive staining was observed, the hyphal cells were in the late stage of apoptosis or necrosis [28]. As shown in Figure 5, when the concentration of LEO reached 0.10%, the hyphae appeared PI positive, and when the concentration gradually increased, the number of PI-stained hyphae gradually increased. These results showed that when the concentration of LEO/NE was gradually increased, apoptosis or cell death of *Phomopsis* sp. resulted.

To investigate the effects of LEO/NE on the postharvest decay of kiwifruit caused by *Phomopsis* sp., a preservation experiment was carried out by inoculating *Phomopsis* sp. spores in fresh kiwifruit samples. As shown in Table 2, there was no significant difference (*p* > 0.05) in the viscosity, elasticity, chewiness, soluble solids, or titratable acid of kiwifruit treated with various LEO/NE additions after storage at 25 °C for 12 d. The hardness increased with increasing of LEO concentration, but the increasing range was not significant. One possible reason for this observation is that kiwifruit conduct a kind of climacteric respiration, and the ethylene produced in the batch storage process will affect its maturity and thus modulate the above indicators [6].

### 3.9. Effect of LEO/NE on the Postharvest Decay of Kiwifruit Caused by Phomopsis *sp*.

**Figure 6:**
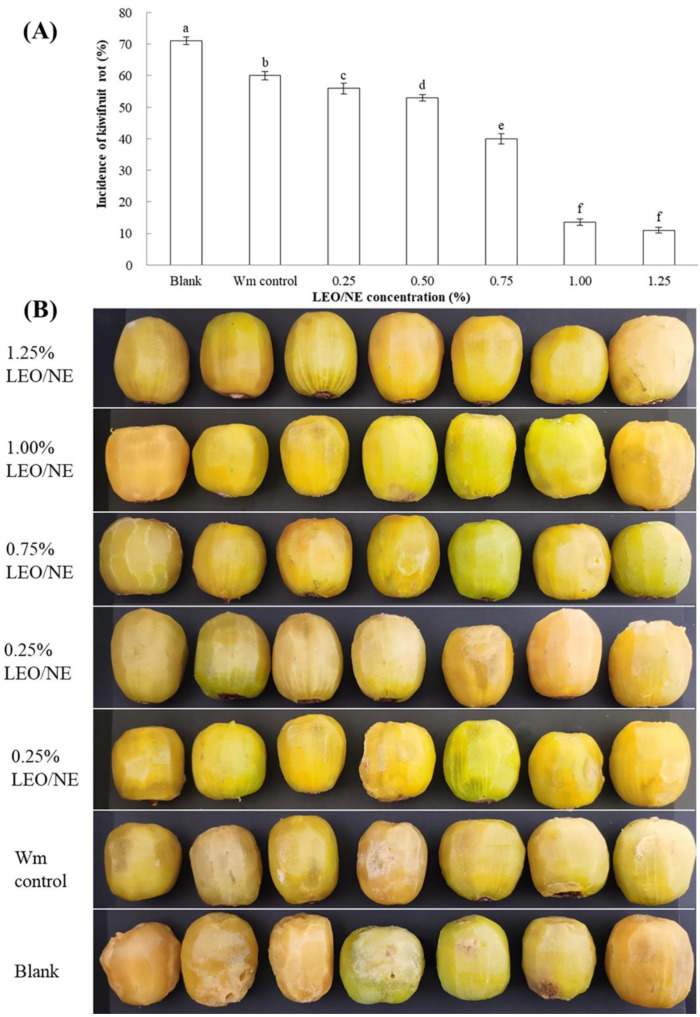
Effect of lemon essential oil nanoemulsion (LEO/NE) on the postharvest decay of kiwifruit caused by *Phomopsis* sp. (**A**) incidence of kiwifruit rot, (**B**) appearance of kiwifruit rot. Different letters above each column of (**A**) indicate significant differences.

As shown in Figure 6A, after 12 d of preservation, the incidence of kiwifruit rot treated with LEO/NE decreased in a LEO dose dependent manner. The increased nanoemulsion concentration was significantly lower than that of the Wm control group (*p* < 0.05). However, the incidence of kiwifruit rot for the nanoemulsion with a 1.25% LEO loading amount was not significantly higher than that of the nanoemulsion with a 1.0% LEO loading amount. These results could also be more intuitively observed from the appearance of fruit. As shown in Figure 6B, the rot symptoms of kiwifruit in the blank group were similar to those in the Wm control group, but the kiwifruit of these two groups were obviously more rotten than those in the LEO/NE group. With the increase in the concentration of the LEO in the emulsion, the extent of kiwifruit rot and the size of the rotted parts significantly decreased (Figure 6B), and the inhibitory effect had not been significantly improved after the loading amount of the nanoemulsion exceeded 1% LEO. These results indicated that the microcapsule treatment of LEO significantly inhibited the rot of kiwifruit caused by *Phomopsis* sp., and the nanoemulsion with a LEO loading amount of 1% showed the best results in statistical terms.

## 4. Conclusions

In the present study, LEO was encapsulated by KGOS to prepare a LEO nanoemulsion (LEO/NE). The LEO/NE showed significant inhibitory effects on the soft rot pathogens of kiwifruit *Phomopsis* sp. compared to that of the wall material control group. The inhibitory effect can be attributed to a direct negative effect on spore germination and mycelial growth of *Phomopsis* sp. by promoting ROS accumulation, intracellular antioxidant enzyme activities, and cell apoptosis. LEO/NE effectively inhibited soft rot development in kiwifruit in a LEO dose dependent manner, and 1% LEO loading amount has a good effect on preventing postharvest decay of kiwifruit. This study indicated that lemon essential oil-based nanoemulsions have great application potential in reducing the postharvest decay of kiwifruit as an efficient green preservative. However, in the future, the research on which chemical components in LEO has the best inhibitory effect need to be further determined.

## Figures and Tables

**Table 1:** Chemical composition and relative percentage of identified compounds in the lemon essential oil.

Number	Compounds	RT (min)	RI	Relative Percentage (%)
1	α-Phellandrene	4.817	997	0.44 ± 0.01
2	α-Pinene	4.925	1005	1.87 ± 0.04
3	Camphene	5.115	1016	0.06 ± 0.00
4	Sabinene	5.375	1031	1.99 ± 0.04
5	β-Pinene	5.449	1035	12.43 ± 0.07
6	7-Methyl-3-methyleneocta-1,6-diene	5.507	1039	1.45 ± 0.02
7	Octanal	5.635	1046	0.05 ± 0.00
8	1,3,6-Octatriene,3,7-dimethyl-, (3E)-	5.73	1052	0.05 ± 0.01
9	α-Terpinene	5.868	1060	0.20 ± 0.01
10	Benzene,1-methyl-2-(1-methylethyl)-	5.956	1065	0.22 ± 0.02
11	D-Limonene	6.047	1070	67.01 ± 0.50
12	(Z)-β-Ocimene	6.165	1077	0.18 ± 0.00
13	γ-Terpinene	6.350	1088	9.69 ± 0.06
14	α-Terpinolene	6.692	1117	0.39 ± 0.02
15	Linalool	6.747	1124	0.08 ± 0.01
16	Nonanal	6.790	1129	0.13 ± 0.00
17	Citronellal	7.344	1198	0.06 ± 0.01
18	α-Terpineol	7.837	1228	0.14 ± 0.02
19	2,6-Octadienal,3,7-dimethyl-, (2Z)-	8.320	1256	0.77 ± 0.03
20	*cis*-Citral	8.610	1273	1.39 ± 0.04
21	2,6-Octadien-1-ol,3,7-dimethyl-, 1-acetate	9.500	1318	0.31 ± 0.01
22	Neryl acetate	9.688	1326	0.21 ± 0.01
23	*cis*-β-Farnesene	10.385	1355	0.19 ± 0.00
24	1,3,6,10-Dodecatetraene,3,7,11-trimethyl-, (3Z,6E)-	10.426	1357	0.30 ± 0.02
25	β-Bisabolene	11.188	1389	0.48 ± 0.03

Values are expressed as mean ± standard error of triplicate analysis. RT: retention time, RI: retention index.

**Table 2:** The hardness, Gumminess, Springiness, Chewiness, soluble solids, and titratable acid of kiwifruit treated by LEO/NE.

	Hardness (g)	Gumminess	Springiness	Chewiness	SSC (%)	TA (%)
Blank	763.87 ± 93.46	38.92 ± 0.63	0.20 ± 0.01	12.11 ± 0.57	12.3 ± 0.216	0.54 ± 0.04
Wm control	760.44 ± 98.71	41.78 ± 1.56	0.37 ± 0.02	15.41 ± 0.25	12.5 ± 0.216	0.49 ± 0.03
0.25% LEO/NE	660.07 ± 85.99	44.49 ± 0.67	0.28 ± 0.01	12.48 ± 0.63	10.0 ± 0.216	0.44 ± 0.05
0.5% LEO/NE	854.63 ± 89.65	41.00 ± 0.42	0.43 ± 0.00	11.51 ± 0.36	11.2 ± 0.287	0.53 ± 0.08
0.75% LEO/NE	915.61 ± 77.27	47.82 ± 0.82	0.31 ± 0.00	13.29 ± 0.29	10.7 ± 0.294	0.48 ± 0.04
1% LEO/NE	928.27 ± 65.84	53.79 ± 1.37	0.39 ± 0.00	12.72 ± 0.25	10.2 ± 0.125	0.48 ± 0.08
1.25% LEO/NE	925.58 ± 87.22	42.04 ± 1.55	0.30 ± 0.02	15.68 ± 0.23	11.3 ± 0.236	0.56 ± 0.05
*p*	0.171	0.777	0.063	0.925	0.913	0.115

## Data Availability

The data presented in this article is available on reasonable request, from the corresponding author.
